# Antimicrobial and anti-inflammatory potential of *Angelica dahurica* and *Rheum officinale* extract accelerates wound healing in *Staphylococcus aureus*-infected wounds

**DOI:** 10.1038/s41598-020-62581-z

**Published:** 2020-03-27

**Authors:** Wan-Ting Yang, Chun-Yen Ke, Wen-Tien Wu, Yi-Hsiung Tseng, Ru-Ping Lee

**Affiliations:** 1 Department of Orthopedics, Hualien Tzu Chi Hospital, Buddhist Tzu Chi Medical Foundation, Hualien, 97002 Taiwan; 2 Department of Nursing, St. Mary’s Medicine, Nursing and Management College, Yi-Lan, 26644 Taiwan; 3 Department of Nursing, Tzu Chi University of Science and Technology, Hualien, 970 Taiwan; 4 Institute of Molecular Biology, National Chung Hsing University, Taichung, 402 Taiwan; 5 Institute of Medical Sciences, Tzu Chi University, Hualien, 97004 Taiwan

**Keywords:** Physiology, Animal physiology

## Abstract

Wound infection is a serious clinical problem, and the most common infection-causing bacteria are *Staphylococcus aureus* and *Pseudomonas aeruginosa*. *Angelica dahurica* and *Rheum officinale* extract (ARE) was reported to accelerate excisional wound healing in rats. In this study, we investigated the therapeutic effects of ARE on bacterial-infected wounds. Thirty Sprague-Dawley rats were divided into three groups: normal saline (NS), ARE, and biomycin ointment (BO). Full-thickness dorsal excisions in all the rats were infected with 10^8^ colony-forming units of *S. aureus*; the treatments were applied once daily for 7 days. Results showed that the residual wound area in ARE group was smaller than those in NS and BO groups. TBCs on wound sites gradually decreased in ARE and BO groups. The body temperature and plasma inflammatory cytokines (TNF-α, IL-6) levels increased after bacterial infection at 24 h in all groups. After treatment, BT and inflammatory cytokines levels decreased in ARE group. Histological observations showed ARE group exhibited earlier scab formation, denser dermal granulation tissue, thicker epidermis, and more angiogenesis markers than the other groups. In conclusion, ARE accelerated wound healing in *S. aureus*-infected wounds. We proposed ARE exhibited potential antimicrobial and anti-inflammatory effects and stimulated angiogenesis, thus improving healing in infected wounds.

## Introduction

Wound infection is a serious problem in clinical practice and causes immune responses and systemic inflammatory response syndrome (SIRS)^1–3^. *Staphylococcus aureus* and *Pseudomonas aeruginosa* are the most common bacterial pathogens found in infected wounds^4,5^. Antibiotics, such as bacitracin, neomycin, and mupirocin, are most commonly used for treating wound infection; these antibiotics are usually administered topically or orally depending on the severity of the infection^6^. However, antibiotic overuse may cause side effects and increase the risk of antimicrobial resistance^7,8^. Notably, antibiotics may delay wound healing^9^. Therefore, new strategies for treating infected wounds are needed.

Wound healing is a complex process involving blood cells, cytokines, and growth factors. Blood glucose levels, leukocyte counts, and levels of proinflammatory cytokines, such as tumor necrosis factor (TNF)-α and interleukin (IL)−6, increase and induce SIRS during the inflammation phase of healing^2,3^. The transforming growth factor (TGF)-β also plays a critical role in accelerating wound healing^10,11^. Angiogenesis is a critical component of wound healing, and the markers are including CD31 and vascular endothelial growth factor (VEGF), in which CD31 is well established for monitoring the vessel density and VEGF is an important signaling molecule involved in angiogenesis^12,13^. Traditional Chinese medicine (TCM) and some herb-derived compounds, such as those prepared using *Aloe vera* and *Calendula officinalis*, have become popular for the treatment of skin lesions^14^. Studies have shown that TCM treatments may provide multiple benefits, including anti-inflammatory, antimicrobial, and cell-stimulatory activities, which collectively promote wound healing^14,15^. A previous study also reported the antimicrobial and anti-inflammatory effects of *Angelica dahurica* and *Rheum officinale* extract (ARE) and its ability to accelerate wound healing in excisional wounds in rats^15^. However, the mechanism underlying wound healing by ARE has not been elucidated thus far. Therefore, in the present study, an excisional wound rat model was used to investigate the effects of ARE on infected wound.

## Results

### Antimicrobial effects and active constituents contained in ARE

**Figure 1:**
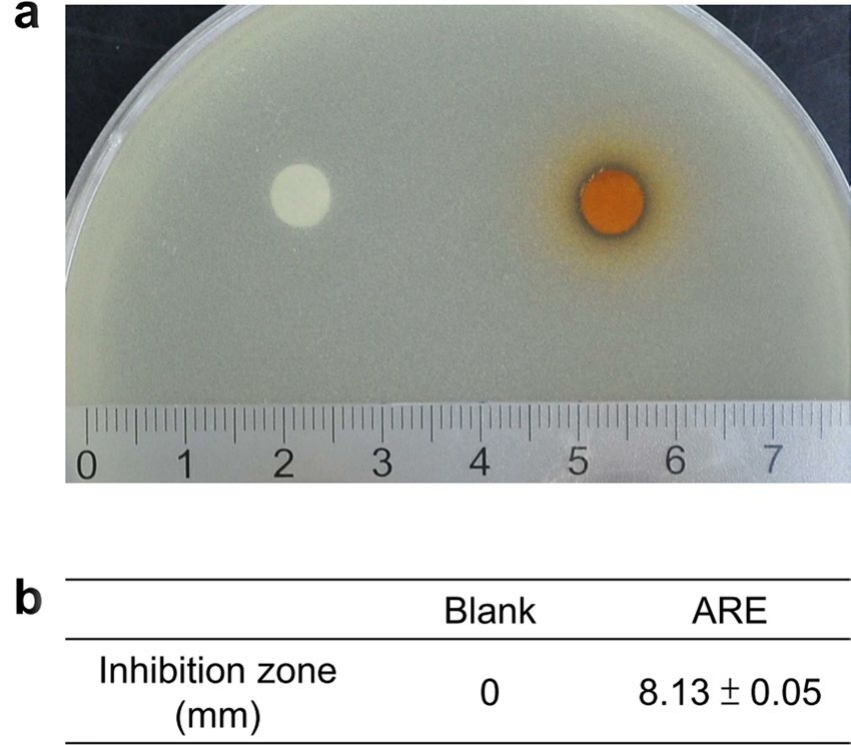
Antimicrobial effects of ARE on *S. aureus* ATCC 29213 by disc diffusion test. **(a)** The inhibition zone of ARE against *S. aureus* ATCC 29213. **(b)** Average diameter (mm) of the inhibition zones. Data are expressed as mean ± standard error of the mean.

The results of disc diffusion indicated that 10 μL of ARE on 6 mm discs (containing 11.02 ± 0.22 μg/disc of solid extract) had an average 8.13 ± 0.05 mm inhibition zone against *S. aureus* (Fig. 1). HPLC analysis revealed that the ARE used in this study contained 32 μg/mL of aloe-emodin, 12 μg/mL of chrysophanol, 36 μg/mL of emodin, 3 μg/mL of physcion, and 95 μg/mL of rhein, while psoralen were not detected.

### Effects of ARE treatment on wound closure

**Figure 2:**
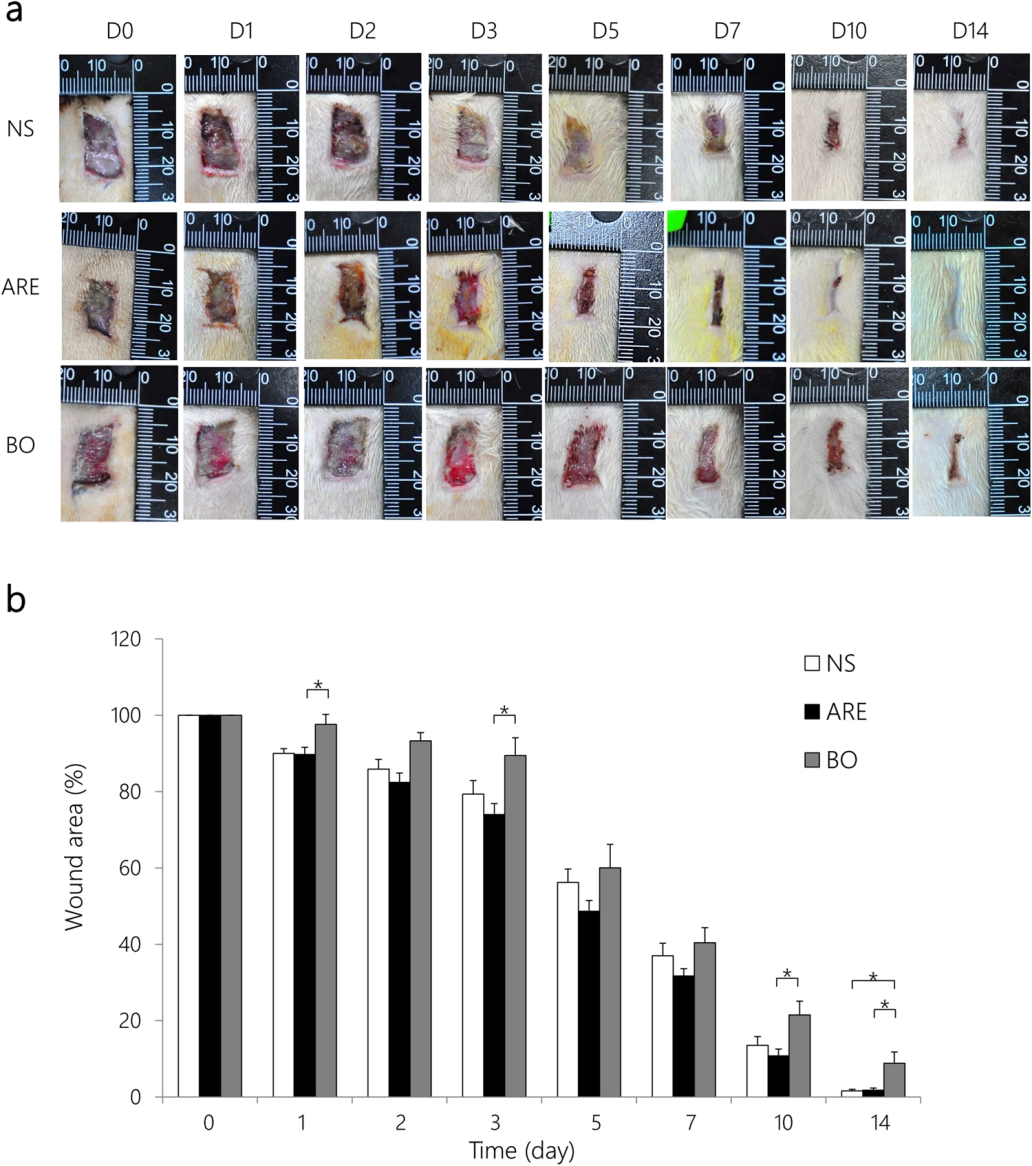
Macroscopic analysis of the wound healing process. **(a)** Macroscopic view of *S. aureus*-infected wound treated with normal saline (NS), *Angelica dahurica* and *Rheum officinale* extracts (ARE), and Biomycin ointment (BO) at days 0, 1, 2, 3, 5, 7, 10, and 14 after treatment. **(b)** Changes in wound sizes in each group at days 0, 1, 2, 3, 5, 7, 10, and 14 after treatment; expressed as a percentage of the initial wound area. Data are expressed as mean ± standard error of the mean, *P < 0.05.

The photographs of the wound sites (Fig. 2a) and the percentage of residual wound areas (Fig. 2b) after wound healing on days 0, 1, 3, 5, 7, 10, and 14 are shown in Fig. 2. The mean of residual wound area in the ARE group was 73.99% on day 3, which was significantly smaller than that in the BO group (89.47%, *P* < 0.05). On day 5, the healing wounds were already half closed in the ARE group (residual wound area 48.67%), whereas the residual wound areas in the NS and BO groups were 56.21% and 60.02%, respectively. On day 7, the residual wound area (31.71%) in the ARE group was also smaller than that in the BO group (40.41%), however, there was no statistical significance between groups. After 14 days of treatment, the residual wound area in the ARE group was 1.74% (*P* < 0.05), whereas that in the BO group was 8.81%. Overall, the residual wound area in the ARE group was smaller those in the NS and BO groups after treatment.

### Total bacterial counts on wound site

**Figure 3:**
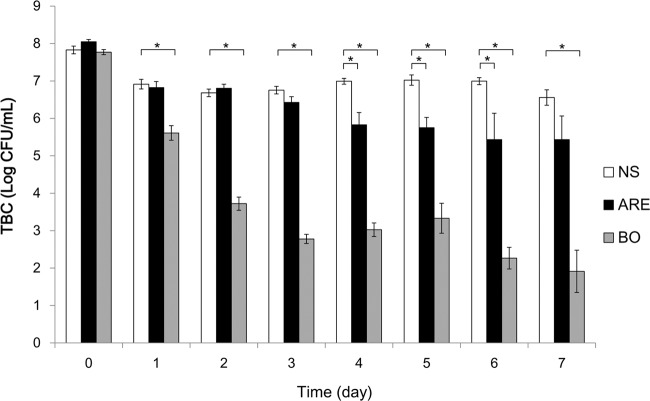
Total bacterial count on wound site. The total bacterial counts on wound sites were recorded using tryptic soy agar plates, with the plates incubated at 37 °C for 24 h. Data are expressed as mean ± standard error of the mean, *P < 0.05.

A TBC of approximately 8 log CFU/mL (10^8^ CFU/mL) was detected in the wound site on the day after infection (day 0). After treatment, the TBCs on the wound sites gradually decreased in the ARE and BO groups (Fig. 3). On day 7, the TBCs on the wound sited decreased to 6.56 ± 0.21, 5.43 ± 0.63, and 1.91 ± 0.56 log CFU/mL in the NS, ARE, and BO groups, respectively. In general, the BO group had a lower TBC than the other groups.

### Body temperature

**Figure 4:**
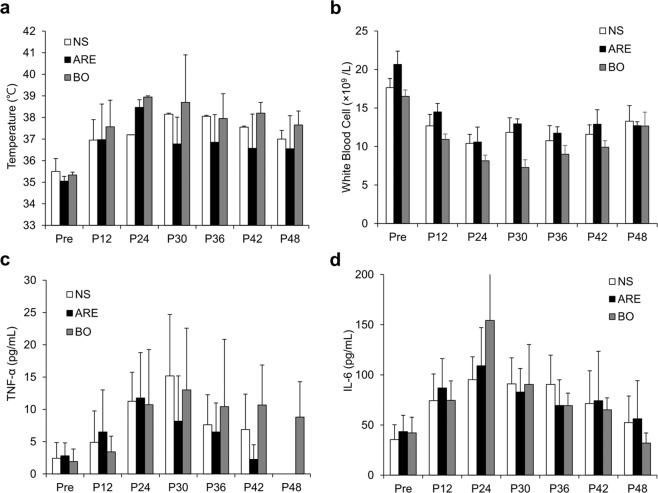
Time course of changes in inflammatory markers after wound infection and after treatments. Each treatment was applied at p24. **(a)** Body temperature, **(b)** white blood cell (WBC) count **(c)** plasma TNF-α level **(d)** plasma IL-6 level. Data are expressed as mean ± standard error of the mean, *P < 0.05.

The average BTs were 35.0 °C–35.5 °C at the beginning of the study (pre) in the NS, ARE, and BO groups. At 24 h after *S. aureus* infection, the BT gradually increased in the three groups. After 24 h of infection, the BTs increased to 37.2 °C–39.0 °C (Fig. 4a). The treatments were first applied at 24 h. After treatment, the BTs decreased in the ARE and BO groups; whereas the BT in the NS group (untreated group) remained high. In the ARE group, at 12 h after treatment (p36), the BT decreased from 38.5 °C to 36.9 °C and was 36.6 °C after 24 h of treatment (p48). In the BO group, the BT decreased from 39.0 °C to 38.0 °C after treatment for 12 h (p36) and was 37.7 °C after treatment for 24 h (p48) (Fig. 4a).

### The WBC counts and plasma TNF-α, IL-6 levels

The average WBC counts were 16.51–20.66 × 10^9^/L before wounding in the NS, ARE, and BO groups. After 24 h of infection, the WBC counts gradually decreased in the NS, ARE, and BO groups and were 8.15–10.58 × 10^9^/L at p24. After treatment, the WBC counts gradually increased until p48 (12.66–13.28 × 10^9^/L) (Fig. 4b). The average plasma TNF-α levels were 2.0–2.8 pg/mL in the NS, ARE, and BO groups before wounding. After infection, the TNF-α levels gradually increased. After 24 h of infection, the TNF-α levels increased to 10.71–11.75 pg/mL. After 6 h of treatment, the TNF-α levels in the ARE group decreased to 8.17 pg/mL (p30), whereas the TNF-α levels continued to increase in the other two groups (15.17 and 13.0 pg/mL in the NS and BO groups, respectively) (Fig. 4c). The changes of plasma IL-6 levels were similar as TNF-α changes, which increased after infection and were observed with a highest level after 24 h of infection, and then gradually decreased after treatment (Fig. 4d).

### Histological observations of wound areas

**Figure 5:**
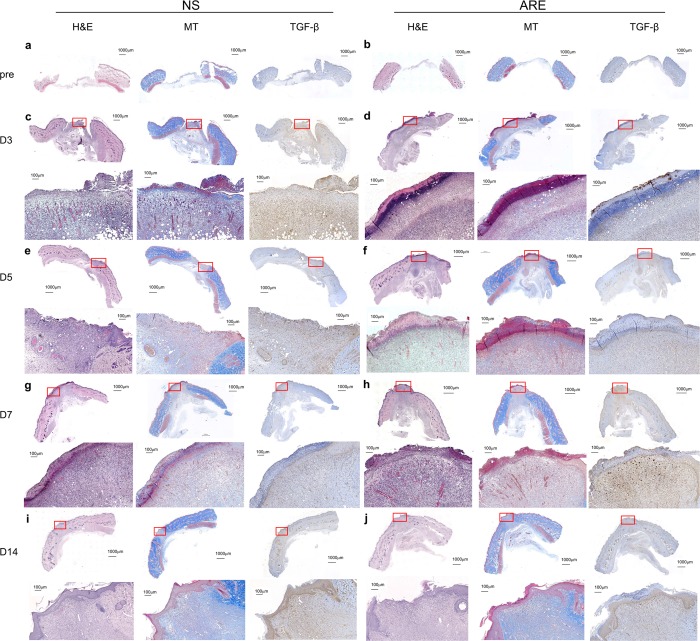
Histological view of skin wound on pre (before treatment) and days 3, 5, 7 and 14 after treated with NS and ARE. Stained with hematoxylin and eosin (H&E), Masson’s trichrome (MT), and TGF-β immunohistochemistry (IHC) staining.

**Figure 6:**
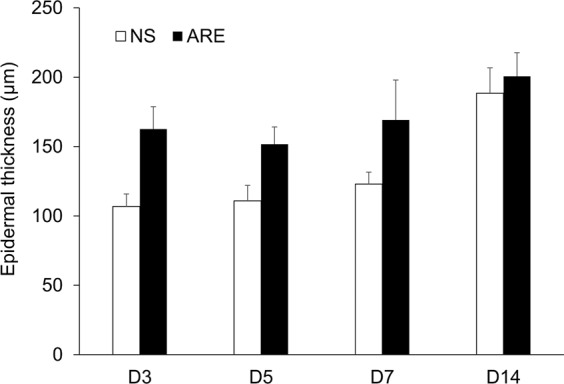
The thickness of epidermis of healed skin at days 3, 5, 7 and 14 after treatment. Data are expressed as mean ± standard error of the mean.

**Figure 7:**
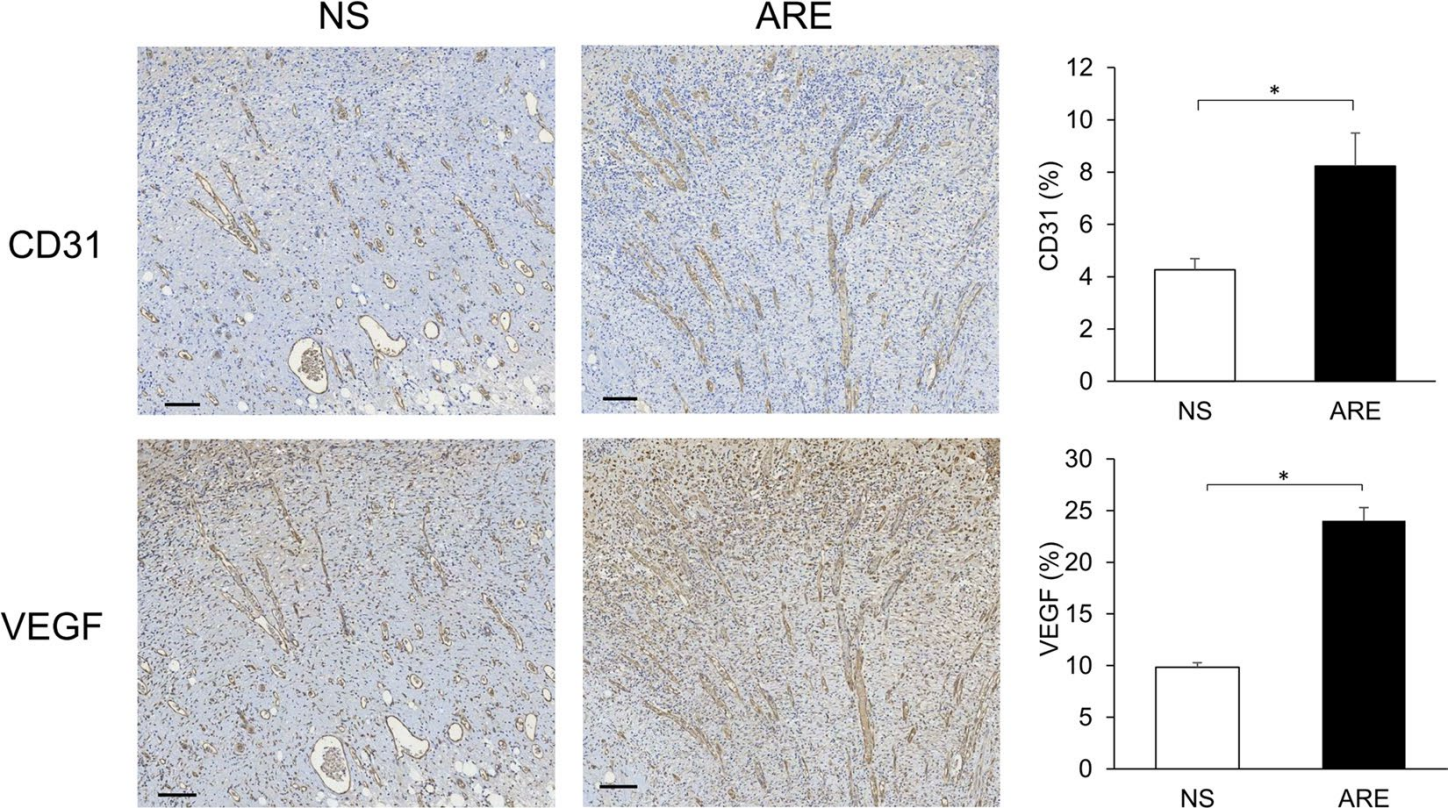
IHC staining for angiogenesis markers CD31 and VEGF of skin wound on day 7 after treatment, and the quantification of CD31 and VEGF positive cells. Scale bars: 100 μm; error bars indicate standard error of the mean, *P < 0.05.

The skin biopsies of the wound sites were investigated before treatment (pre) and on days 3, 5, 7, and 14 after treatment to assess the extent of healing. The histological views of the full-thickness excisional wound sites are shown in Fig. 5a,b. On day 3 after treatment, the dermis surrounding the wound site in the NS group was thicker than that in the ARE group, which showed swelling and extrusion of the skin (Fig. 5c,d). Furthermore, on day 3, a loose and damaged dermis and hemorrhage were observed in the dermis layer under the wound site in the NS group, whereas scab formation, a dense dermis, and a relatively high number of hyperchromatic cells were observed in the ARE group (Fig. 5c,d). On day 5, scab formation was not observed in the NS group, and the gaps around the wound site were filled (Fig. 5e). Furthermore, on day 5, the dermis was also denser in the ARE group than in the NS group (Fig. 5e,f). The width of the wound in the ARE group on day 5 was significantly less than that on day 3 (Fig. 5c,e). Scab formation was observed in the NS group on day 7 (Fig. 5g), and the dermis was denser on day 7 than it was on days 3 and 5 (Fig. 5c,e). Masson’s trichrome staining results showed that collagen-rich fibrotic regions (blue area) regrew inward to the wound site in the ARE group (Fig. 5d,f,h). Immunohistochemistry (IHC) results showed that more TGF-β1 was detected at the wound site in the NS group on day 3 (Fig. 5c) and progressively decreased on days 5 and 7 (Fig. 5e,g). More TGF-β1 was detected at the wound site on day 7 in the ARE group than in the NS group (Fig. 5g,h). Regeneration of the epidermis was observed in the NS and ARE groups on day 14, without significant difference between groups (Fig. 5I,j). In additional, the thickness of epidermis in the ARE group was 162.5 ± 16.21μm, 151.67 ± 12.49, and 169.17 ± 28.71 μm on days 3, 5 and 7, respectively, which were thicker than those in the NS group (106.86 ± 9.04, 110.83 ± 11.21, 122.94 ± 8.57 μm on days 3, 5 and 7, respectively) (Fig. 6). Notably, more new blood vessel formation was observed in the ARE group on day 7 (Fig. 5h), which possibly suggested that ARE treatment stimulates angiogenesis during wound healing. Therefore, the angiogenesis markers, CD31 and VEGF, were evaluated by IHC to confirm the speculation. Results indicated that, more CD31 and VEGF were detected in ARE group on day 7, and the amount were approximately two times more than those in NS group (*P* < 0.05, Fig. 7).

## Discussion

The ethanol extracts of *A. dahurica* and *R. officinale* (ARE) were reported to exhibit antibacterial and anti-inflammatory effects *in vitro* as well as promote wound healing in a rat model of excisional wounds^15^. In this study, we demonstrated that ARE is also an appropriate alternative medication for treating wound infection. The wound healing results showed that the residual wound area in the ARE group was smaller than that in the BO group on days 1, 3, 5, 7, 10, and 14 after treatment (Fig. 2). In addition, the residual wound area in the ARE group was smaller than that in the NS group on days 2, 3, 5, 7, and 10 after treatment (Fig. 2b), although the difference between the two groups was not statistically significant. The pattern of wound healing observed in this study is consistent with that reported by previous studies; the wounds in the ARE group healed better than those in the NS group did on days 3–7, revealing the effects of ARE in the inflammatory phase^15^. To verify whether the healing effect of the infected wound resulted from the antimicrobial effect of ARE, the TBCs on wound sites after treatment were measured for 7 days. The TBCs on wound sites gradually decreased in the ARE and BO groups after treatment. After 7 days of treatment, the average TBCs on wound sites decreased from the initial 8 to 5.4 and 1.9 log CFU/mL in the ARE and BO groups after 7 days of treatment, respectively. The results showed that the TBCs on the wound site after BO treatment were significantly lower than those after NS and ARE treatment (*P* < 0.05) (Fig. 3); thus, BO exhibited a higher antimicrobial activity than ARE. The major constituent of BO is neomycin, an antibiotic that is used to treat skin bacterial infections^16^; however, the extent of wound closure in the BO group was less than those observed in the NS and ARE groups (Fig. 2b). This finding was consistent with previous observations^9,15^. The wound sites treated with antibiotic ointment exhibited slower healing rates than those treated with the NS group, suggesting that antibiotic use may delay wound healing. Ito *et al*. reported that, although local or systemic antibiotic treatments reduce bacterial loads on the skin, the wound repair was delayed^9^. Toll-like receptors recognize invasive microorganisms by binding to pathogen-associated molecular patterns (PAMPs) and activating the innate immune system, thereby initiating a protective response^17^. An unappreciated side effect of antibiotic therapy was also demonstrated that, antibiotic use reduced the PAMP-dependent activation of the innate immune system by reducing the bacterial load on wounds; thus, antibiotic use adversely affected the rate of wound healing^9^. Although the antimicrobial effects of ARE were not comparable to those of the antibiotic, the wound closure results in the ARE group were superior to those in the BO group. The ARE contained polysaccharides, which was demonstrated to promote the proliferation of rat skin cells *in vitro*^18,19^. ARE also contained rhein, emodin, aloe-emodin, chrysophanol and physcion, which possessed antimicrobial effect against *S. aureus*, among them emodin also provided to enhance cutaneous wound healing in rats^19–21^. There was a similar finding in our study, compared with the control group, the ARE treatment not only possessed antimicrobial effects, but also promoted wound healing.

Hyperthermia and elevation of plasma TNF-α levels occurred within 24 h of bacterial infection. BTs increased from 35.0 °C–35.5 °C to 37.2 °C–39.0 °C (Fig. 4a), and the levels of plasma TNF-α and IL-6 increased after 24 h of infection in the three groups (Fig. 4c,d). Fever is among the most common systemic clinical signs during infection and is mediated by the release of pyrogenic cytokines such as TNF-α and IL-6^3^. The peptidoglycan and lipopolysaccharide of bacteria increase the levels of TNF-α and IL-6 and cause a cytokine cascade of fever induction^2,3,22^. Furthermore, the WBC count decreased from 16.51–20.66 to 8.15–10.58 × 10^9^/L after 24 h of infection in the three groups; thus, the WBC counts at 24 h after infection were lower than the initial WBC counts (Fig. 4b). These observations indicated that SIRS occurred after 24 h of infection^1^. All the treatments were applied at p24; BTs decreased in the ARE and BO groups at p30, whereas BT remained increasing in the NS group (Fig. 4a). At p30, the plasma TNF-α level decreased in the ARE group, whereas it increased in the other two groups (Fig. 4b). The WBC count gradually increased until p48 without a significant difference among the groups (Fig. 4c). Our previous results demonstrated that ARE reduced the expression of TNF-α released by LPS-induced THP-1 cells^15^; in this study, the plasma TNF-α decreased after ARE treatment. Besides, TNF-α is reported to exert a detrimental effect on healing, which may inhibit wound re-epithelialization^11^. These results showed that, after ARE treatment, both BT and TNF-α levels decreased, thus suggesting that the anti-inflammatory effects of ARE were involved in the treatment effect.

Scab formation alters the pattern of epidermal cell migration during wound healing^23^. Histological observations in this study showed that scab formation occurred on days 3 and 7 in the ARE and NS groups, respectively (Fig. 5). A previous study showed that the scabs were thicker in the ARE group than in the NS group in an excisional wound healing model^15^; these data suggest that ARE treatment accelerated scab formation during wound healing. A dense dermis was observed in the ARE group, which revealed that, more granulation tissue were developed after ARE treatment than other treatments. Our results were consistent with previous research findings; Zhang *et al*. reported that ethanolic extracts of *A. dahurica* promoted granulation in diabetic wound healing models^24^. Angiogenesis is a critical component of wound healing^12,13^. In this present study, more new blood vessel formation was observed in the ARE group than in other groups (Fig. 5); furthermore, more CD31 and VEGF were detected in ARE group on day 7, and approximately two times more than those in NS group (Fig. 7), which indicated that ARE treatment stimulates angiogenesis during wound healing. This finding is also consistent with those of previous studies on ethanolic *A. dahurica* extract; it induced angiogenesis *in vivo* in rat diabetic wounds and *ex vivo* in rat aortic ring assays^24^. TGF-β plays a critical role in wound healing, which is produced by platelets, keratinocytes, macrophages, lymphocytes, and fibroblasts^11^. TGF-β1 is involved multiple processes in wound healing, including inflammation, granulation tissue formation, angiogenesis stimulation, re-epithelialization, and extracellular matrix formation and remodeling; therefore, it improves wound healing^10,11,25^. Histological results showed that TGF-β1 was detected at the wound site on day 3 in the NS group (Fig. 5c), but it gradually decreased by days 5 and 7 (Fig. 5e,g). By contrast, less TGF-β1 was detected at the wound site on day 3 in the ARE group, and more TGF-β1 was detected at the wound site on day 7 (Fig. 5h). Several studies have indicated that TGF-β1 regulate wound healing, particularly during the later stages, which involve keratinocyte proliferation, granulation tissue formation, and epithelialization^11,25^. Much TGF-β1 was recruited to the wound site on day 7 in the ARE group, demonstrating the recruitment of TGF-β1 to the wound site at a late stage in wound healing. The results showed that ARE promoted wound healing in the ARE-treated group. According to that TNF-α has been reported to exert an antagonistic activity against the effects of TGF-β through inhibition of TGF-β/Smad signaling^25–27^, our results speculated that the anti-inflammatory effect of ARE reduced the TNF-α level, thereby altering the recruitment of TGF-β1 to the wound site in the inflammatory phase. Consequently, more TGF-β1 were observed at the wound site on day 7 in the ARE group and promoted wound healing.

**Figure 8:**
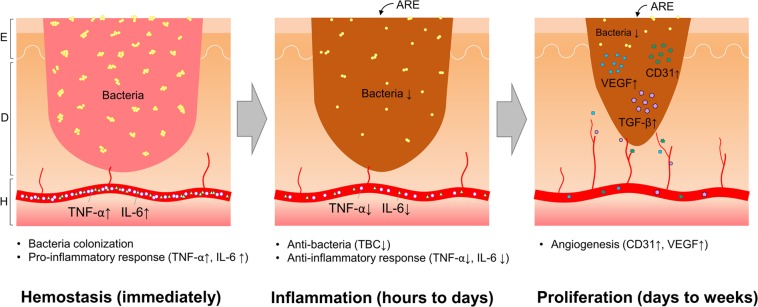
Possible mechanism of ARE in the inflammatory phase and proliferative phase of wound healing. E: epidermis, D: dermis, H: hypodermis.

This study has some limitations. First, we did not examine downstream Smad signaling to support the effects of ARE on TGF-β; we only speculated this from previous research. Second, we compared the histology of the wound site in the ARE group with that in the control group; however, the BO group was not analyzed. Third, no vehicle group was used in this experiment. Only a control group was used, which might have caused us to overlook the inference of the solvent that was used for the ARE group. In conclusion, ARE accelerated wound healing in *S. aureus*-infected wounds. We propose that ARE exhibits antimicrobial and anti-inflammatory effects during the early stages of healing and stimulates angiogenesis, proliferation, and epithelialization during the later stages of healing; therefore, it can improve wound healing (Fig. 8).

## Materials and Methods

### Preparation of ARE

Extracts of *A. dahurica* and *R. officinale* were prepared as described previously^15^. The Chinese medicinal powders of *A. dahurica* and *R. officinale* were purchased from Hou-Chuia Biopharm Co., Ltd., Tainan City, Taiwan. Initially, 50 g of each powder was individually added to 400 mL of 70% ethanol, and the mixture was heated at 70 °C for 24 h. After extraction, the decoction was centrifuged at 10,000 × *g* for 15 min, and the supernatants were concentrated under reduced pressure to remove all the ethanol. The extracts of *A. dahurica* and *R. officinale* were autoclaved, mixed together in equal volumes, and designated as ARE.

### Quantification of the active constituents in ARE

Quantification of the possible active constituents in ARE, aloe-emodin, chrysophanol, emodin, physcion, rhein, and psoralen, was provided by Herbiotek Co., Ltd., New Taipei City, Taiwan. Twenty milliliter of ARE was filtered through 0.22-μm membranes before analyzed. The Waters HPLC system (Milford, Massachusetts, USA) included Waters 600 pump system, Waters 2996 photodiode array detector, Waters 717 plus autosampler, and Sugai U-620 column oven (Wakayama City, Japan). Cosmosil 5C18-MS-II reversed phase column (5 μm, 4.6 mm × 250 mm, Nacalai Tesque, Japan) equipped with LiChrospher RP-18 end-capped guard column (5 μm, 4.0 mm × 10 mm, Merck, Germany) was used as the stationary phase. The gradient elution was consisted of 10 mM phosphate buffer, acetonitrile, and water. The flow rate was 1 mL/min, and the column temperature was maintained at 35 °C. UV 246 nm was used for detection of psoralen (a furanocoumarin isomer), with a retention time of 14.5 min. UV 270 nm was used for detection of aloe-emodin, rhein, emodin, chrysophanol, and physcion, with a retention time of 89.1, 94.1, 107.4, 114.9, and 118.1 min, respectively. Data are presented as the concentration (μg/mL) of each constituent in ARE.

### Antimicrobial susceptibility test

Disc diffusion test was performed according to the CLSI standard to test the antimicrobial effect of ARE. The method was followed consistent with our previous study^15^. The *S. aureus* ATCC 29213 (Bioresource Collection and Research Center, BCRC, Hsinchu, Taiwan) was cultured in Tryptic Soy Broth (Becton, Dickinson and Company, USA) at 37 °C until log phase and the turbidity was adjusted with the broth to achieve 0.5 McFarland standard (approximately 10^8^ CFU/mL). The autoclaved 6 mm diameter filter paper discs (Advantec Grade Number 1) were soaked with 10 μL of ARE (approximately 11.02 ± 0.22 μg/disc of solid extract) and dried at room temperature. The inoculums (~10^7^ cells) were spread onto Mueller-Hinton agar (Becton, Dickinson and Company, USA), and then the disks were placed onto the agar. Discs containing distilled water were used as negative control (blank). The results were expressed as the means ± SE of diameters (mm) of the inhibition zone scored after overnight incubation.

### Animals

Thirty healthy male adult Sprague Dawley rats (age: 8–10 weeks, weight: 300–350 g) were purchased from the National Laboratory Animal Center (Taipei, Taiwan). The rats were housed in standard cages in a humidity- and temperature-controlled room (55% ± 15%; 22 °C ± 1°C) with 12-h light–dark cycles and received standard amounts of food and water. This study protocol was approved by the Institutional Animal Care and Use Committee of Tzu Chi University (IACUC No: 104084). All experiments were performed in accordance with relevant guidelines and regulations of IACUC.

### Model of excisional wound with bacterial infection

The rats were anesthetized through administration of inhaled isoflurane. When the rats were under anesthesia, a polyethylene catheter (PE-50) was inserted into the right femoral artery for collecting blood samples; subsequently, a full-thickness excisional skin wound (area: 20 × 10 mm^2^, depth: 2 mm) was created on the dorsum of each rat. After creating the wound, 10^8^ colony-forming units (CFU) of *S. aureus* were inoculated into the wound area, and the wound was covered using a piece of sterile gauze of 20 × 10 mm^2^ for 24 h to generate a wound infection model. After the operation was completed, the rats were placed individually in metabolic cages (Shingshieying Instruments, Hualien, Taiwan) for 48 h, and a rectal probe was inserted into the rectum of the rats to record the body temperature (BT) continuously using a temperature monitoring and recording instrument (MV2000, Yokogawa Electric Corporation, Tokyo, Japan). After 48 h of recording and sampling BT, the rats were housed individually in standard cages.

### Wound treatment and measurement

The rats were randomly divided into three groups: NS (control group, treated with normal saline), ARE (treated with ARE), and BO (treated with biomycin ointment). After 24 h of infection, the gauze pieces were removed, and the wounds were cleaned gently with sterile NS swabs. Approximately 0.2 mL of NS, ARE, and BO (CBC Biotechnological & Pharmaceutical Co., Ltd. New Taipei City, Taiwan) were applied topically to the wounds, and the wounds were covered with new sterile gauze pieces and bandaging. The treatments were applied once per day for 7 days. Photographs of the wounds were recorded using a digital camera (Nikon D70, Tokyo, Japan) once on the day before the treatment, and the wound area was recorded using ImageJ software (National Institute of Health, United States). The percentage of the residual wound area after healing was calculated using the following formula: *T*_*N*_/*T*_0_ × 100%. In the formula, *T*_0_ represents the initial wound area on the day after infection, and *T*_*N*_ represents the residual wound area on day *N* after treatment.

### Total bacterial count on the wound site

The gauze pieces (20 × 10 mm^2^) removed from the wounds were transfered into eppendorf tubes containing 1 mL of NS. The samples were serially diluted and spread on tryptic soy agar (TSA) plates. The plates were incubated at 37 °C for 24 h. The total number of colonies on the TSA plates was counted. Total bacterial counts (TBCs) on the wound site were represented as CFU/mL.

### Blood sample analysis

Blood samples (0.8 mL) were obtained from the femoral artery before wounding (pre) and at 12, 24, 30, 36, 42, and 48 h after infection (p12, p24, p30, p36, p42, and p48). The white blood cell (WBC) counts in the blood samples were measured using the Urit 2900 Vet Plus hematology analyzer (Diamond Diagnostics Inc., MA, USA). The blood samples were then centrifuged at 700 × *g* for 10 min at 4 °C. The plasma samples were obtained to measure the TNF-α level using enzyme-linked immunosorbent assay kits (R&D System, Minneapolis, MN, USA). The assay was performed according to the manufacturer instructions, and the resulting solutions were analyzed by measuring the absorbance values at 450 nm using a Dynex MRX II microplate reader (Chantilly, VA, USA).

### Histological examination of the wound area

Skin biopsies of the wound sites were obtained at days 3, 5, and 7. The specimens were fixed using 4% buffered formaldehyde for 24 h, embedded in paraffin after dehydration, and cleared using an ethanol series to xylene. The paraffin blocks were cut into 3-μm tissue sections and mounted on microscopic slides for staining. The sample slides were stained using hematoxylin and eosin (H&E) for general morphology, whereas Masson’s trichrome (MT) staining was used for observing collagen fibers. Immunohistochemistry (IHC) was performed using TGF-β1, CD31, and VEGF antibodies. The tissue sections were deparaffinized, rehydrated, treated with peroxidase reagent for 20 min at room temperature, boiled in citrate buffer (pH 6.0) for 30 min, and blocked using an ImmunoBlock solution for 1 h at room temperature. The tissue sections were then incubated with antibodies (1:200 dilution; BioWorld Inc., Visalia, CA, USA) for 4 h at 37 °C. The sections were treated using a rabbit probe with horseradish peroxidase (HRP) labeling (Toson Co., Ltd., Hsinchu, Taiwan) for 30 min at room temperature, stained using 3,3′-diaminobenzidine brown (1:50 dilution; Toson), and counterstained using hematoxylin. After dehydration, the sections on the slides were covered with coverslips, and the results were examined microscopically. The quantification of IHC was evaluated using ImageJ software (National Institute of Health, United States).

### Statistical analysis

Statistical analysis was performed using one-way analysis of variance (ANOVA) followed by Scheffe’s method with IBM SPSS software version 22 (SPSS Inc., IBM, Chicago, IL, USA). All data are presented as mean ± standard error of the mean; *P* values less than 0.05 were considered statistically significant.
